# Self-induced parametric amplification arising from nonlinear elastic coupling in a micromechanical resonating disk gyroscope

**DOI:** 10.1038/srep09036

**Published:** 2015-03-12

**Authors:** Sarah H. Nitzan, Valentina Zega, Mo Li, Chae H. Ahn, Alberto Corigliano, Thomas W. Kenny, David A. Horsley

**Affiliations:** 1 University of California, Davis, CA, USA; 2 Politecnico di Milano, Milan, Italy; 3 Stanford University, Stanford, CA, USA

## Abstract

Parametric amplification, resulting from intentionally varying a parameter in a resonator at twice its resonant frequency, has been successfully employed to increase the sensitivity of many micro- and nano-scale sensors. Here, we introduce the concept of self-induced parametric amplification, which arises naturally from nonlinear elastic coupling between the degenerate vibration modes in a micromechanical disk-resonator, and is not externally applied. The device functions as a gyroscope wherein angular rotation is detected from Coriolis coupling of elastic vibration energy from a driven vibration mode into a second degenerate sensing mode. While nonlinear elasticity in silicon resonators is extremely weak, in this high quality-factor device, ppm-level nonlinear elastic effects result in an order-of-magnitude increase in the observed sensitivity to Coriolis force relative to linear theory. Perfect degeneracy of the primary and secondary vibration modes is achieved through electrostatic frequency tuning, which also enables the phase and frequency of the parametric coupling to be varied, and we show that the resulting phase and frequency dependence of the amplification follow the theory of parametric resonance. We expect that this phenomenon will be useful for both fundamental studies of dynamic systems with low dissipation and for increasing signal-to-noise ratio in practical applications such as gyroscopes.

Nano- and micromechanical resonators with high quality factor are exquisitely sensitive detectors for weak signals and are particularly widely used in the detection of weak forces in applications such as inertial sensing, atomic force microscopy, and gravimetric sensors. In these applications, mechanical pre-amplification of the force signal is appealing because it reduces the impact of secondary detection noise^1^,^2^ and may allow quantum-limited measurements^3^,^4^. In a conventional linear resonator, setting the input signal's frequency equal to the resonator's natural frequency maximizes the sensitivity, which is proportional to the quality factor, *Q*. Because there are physical limits on the achievable *Q*, various schemes have been proposed to exploit nonlinear mechanisms that might afford additional amplification^5^,^6^,^7^,^8^,^9^. Among these, parametric amplification, in which the resonator's stiffness is modulated at twice its oscillation frequency^10^, can be noise free down to a quantum-mechanical level^11^ and has been shown to result in useful pre-amplification. Parametric amplification also exhibits interesting phenomena such as phase-dependent amplification and thermomechanical noise squeezing^10^, and quality factor enhancement independent from bandwidth^12^. Parametric resonances have also been used to improve frequency noise^13^,^14^, increase the sensitivity of atomic force microscopy (AFM) microcantilevers^10^ Lorentz force magnetometers^15^ and micro-gyroscopes. Gyroscopes using externally-applied parametric amplification to separate drive and sense frequencies, to increase drive-axis bandwidth, or to amplify the Coriolis force have been explored^16^,^17^,^18^,^19^,^20^,^21^ however, despite two decades of research surrounding parametrically-amplified resonators, parametric stiffness variation has always been intentionally induced by external means. Here, we introduce the concept of self-induced parametric amplification, which arises naturally as a result of gyroscope operation. Nonlinear coupling between the gyroscope's two modes introduces parametric amplification of the Coriolis force without the need for externally-applied parametric pumping.

Parametric amplification occurs when two modes consisting of the signal (at frequency *ω*) and an “idler” (at frequency *ω_i_*) are coupled through some nonlinearity via a pump input (at frequency *ω_p_* = *ω* + *ω_i_*). In micromechanical devices, degenerate parametric amplification (*ω_i_* = *ω*) is typically implemented using a pump input to modulate the resonator's stiffness at 2*ω* either electrostatically^17^,^22^,^23^, thermally^24^, or through piezoelectric stiffness modification^25^,^26^. However, some micromechanical resonators naturally exhibit degenerate vibration modes, a fact that is fundamental to the operation of sensors such as micromechanical gyroscopes. Less well-known is the fact that nonlinear elastic behavior arising from both geometric and material effects can result in nonlinear coupling between these modes. In this Article, we describe self-induced parametric amplification arising from nonlinear coupling between the degenerate elliptical vibration modes of a silicon disk resonator. The primary mode's vibration at frequency *ω* creates a time-varying stress field that in turn modifies the stiffness of the secondary mode. Because the stress field is two-fold symmetric, the stiffness modulation occurs at 2*ω*, resulting in self-induced parametric amplification of the secondary mode that is phase-locked to the primary mode's vibration signal. The immediate practical application of this phenomenon is to micromechanical gyroscopes, wherein driven oscillation of a primary vibration mode results in Coriolis force on a secondary degenerate vibration mode when the device rotates. Because the Coriolis force is also phase-locked to the driven oscillation velocity, self-induced parametric amplification can dramatically increase the gyroscope's sensitivity to angular rotation rate.

## Results

### Device and Operation

**Figure 1:**
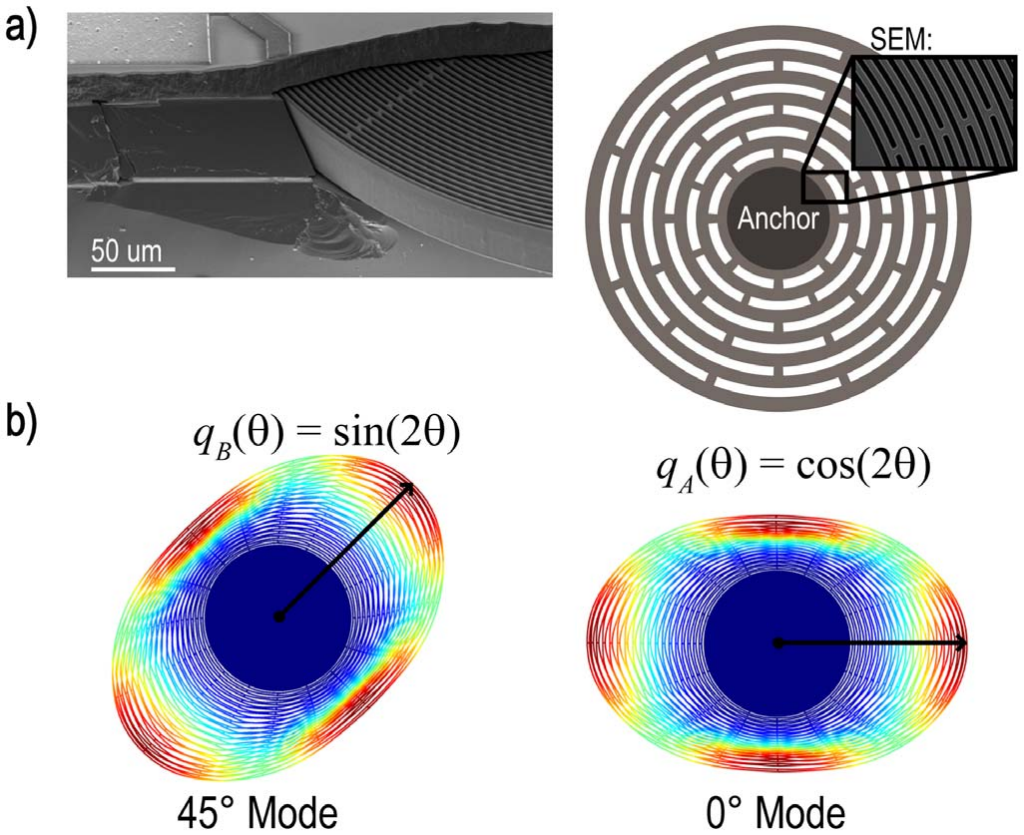
Disk Resonator Gyroscope (DRG). a) SEM of DRG and drawing showing DRG shape, with inset SEM of rings. b) Orthogonal elliptical mode shapes, with color indicating displacement. Red corresponds to maximum displacement, while blue corresponds to zero displacement.

**Figure 2:**
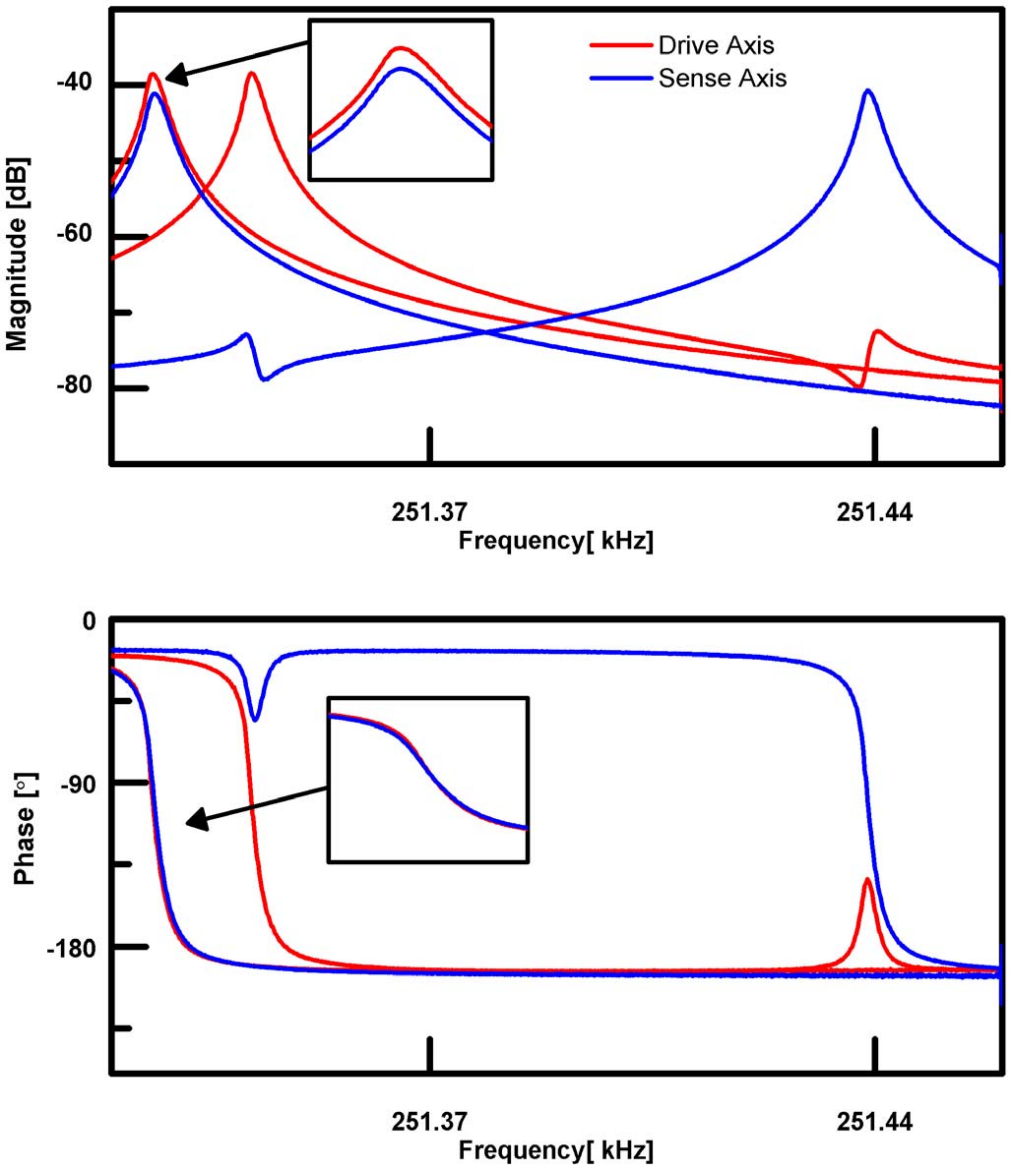
Amplitude and phase response of the two axes of the DRG. Shown before mode-matching (pale lines) and after mode-matching (dark lines and inset figures). The initial frequency mismatch of 90 Hz is reduced to <50 mHz.

The resonator, shown in Fig. 1a, is a 0.6 mm diameter single-crystal silicon slotted disk supported by a central cylindrical post and surrounded by capacitive electrodes used to force and sense vibration^27^. This particular device has attracted interest as a candidate high-performance MEMS gyroscope^28^. The device is vacuum sealed at a pressure near 1 Pa using an epitaxial silicon layer (Supplementary Information). The disk supports a number of radial vibration modes in degenerate pairs whose orthogonal mode shapes are described by sin(*nθ*) and cos(*nθ*) where *n* is the mode index. The *n* = 2 mode shapes used here are shown in Fig. 1b. Fabrication imperfections and crystalline anisotropy, which results in anisotropic elasticity^29^, break the resonator's symmetry, splitting the frequency of the two modes so that they are not perfectly degenerate. Degeneracy is restored via spoke-angle compensation^30^ and electrostatic tuning (Supplementary Information). The frequency response before and after tuning is illustrated in Fig. 2, showing that the initial frequency split *δω*/2*π* = 90 Hz is reduced to less than 50 mHz. Here, a temperature-controlled environment ensures that this tuned condition is maintained, but there are several approaches for maintaining closed-loop tuning of a gyroscope^31^,^32^,^33^.

When operated as a gyroscope, the modes in a degenerate pair are called the drive and sense modes. A phase-locked loop (PLL) maintains sinusoidal oscillation of the drive mode 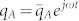, where 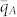 is the amplitude and *ω*/2*π* = 251 kHz is the resonant frequency. When rotation rate, Ω, is applied, this vibration is coupled to the orthogonal sense mode through the Coriolis force, 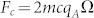 where *m* is the modal mass, *c* is the degree of Coriolis coupling between the two modes, and 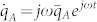 is the velocity of the driven mode (Supplementary Information). A second effect of imperfect symmetry is the introduction of stiffness coupling between the modes, *k_AB_*, producing a force on the sensing mode, 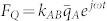, referred to as the quadrature force since its phase is shifted by 90° relative to the Coriolis force. Together, *F_C_* and *F_Q_* are the main forces that excite vibration of the sense mode. The in-phase component of the sense mode vibration *q_B_* is used as a measure of the rotation rate. Typically, the quadrature force, *F_Q_* is regarded as an error source in gyroscopes and is avoided through the use of phase sensitive detection and active cancellation. In a mode-matched gyroscope, the modes are perfectly degenerate and the sense mode amplitude in response to a constant rate input is given by 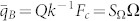, where 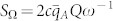 is the rate sensitivity. As a result, a gyroscope's sensitivity to rotation can be increased by maximizing *Q* and operating at large vibration amplitudes 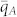, a fact that introduces the need to operate the resonator in the regime where nonlinear mechanical effects are important.

### Parametric Nonlinearities

**Figure 3:**
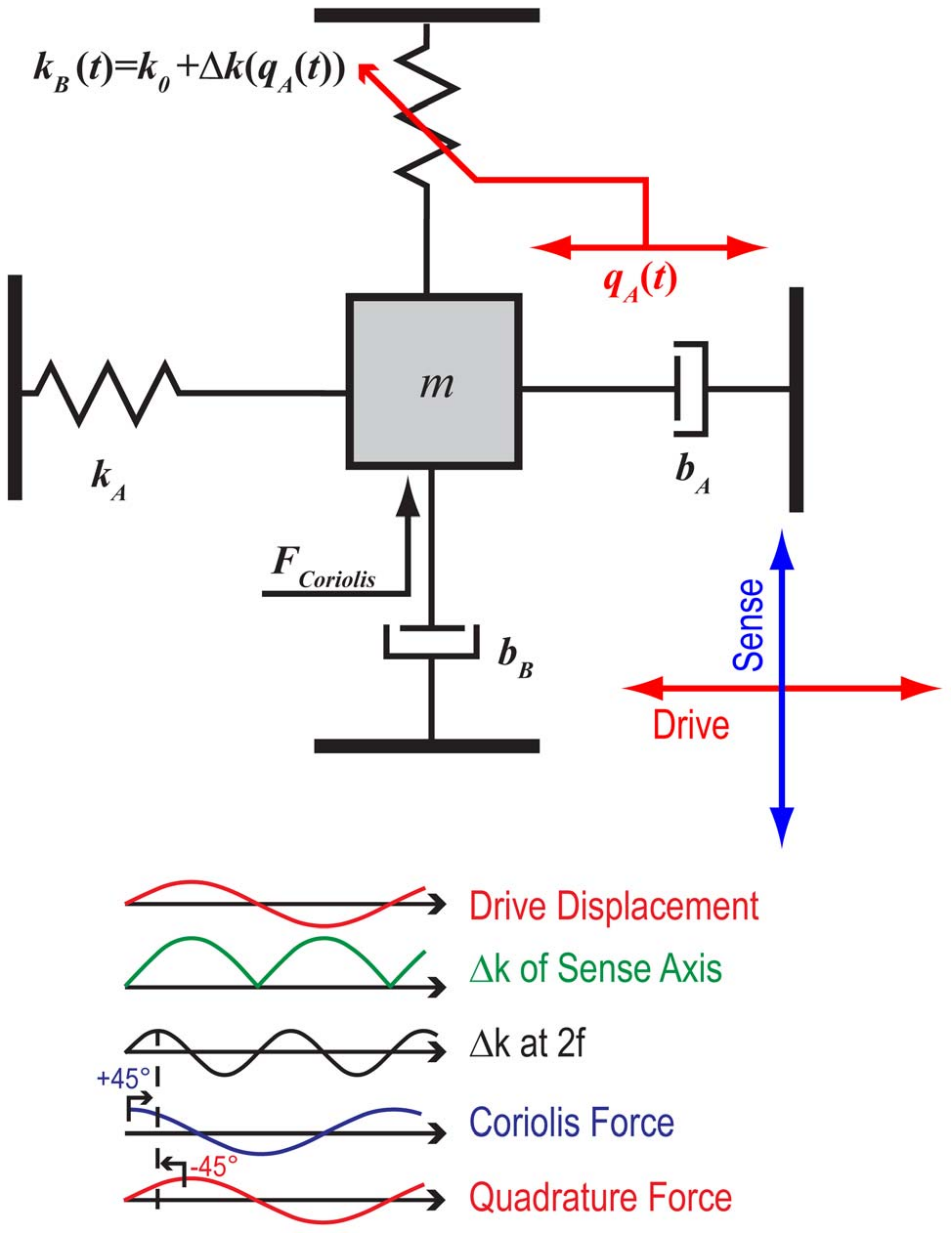
Lumped element model of gyroscope. Due to geometric nonlinearity, displacement of the drive axis (*q_A_*) modulates the stiffness of the sense axis (*k_B_*) at twice the resonant frequency, thus parametrically amplifying Coriolis force and electrostatic inputs to the sense axis. The relative phases of these signals are shown.

At large amplitude, nonlinear mechanical coupling between the two degenerate modes leads to self-induced parametric amplification of the Coriolis force input and causes the rate sensitivity to be dependent on both the amplitude 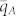 and the phase shift *ϕ* between *q_A_* and the applied forces. A lumped element model for the device (Fig. 3), shows that nonlinear elastic effects cause the driven mode's displacement 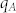 to modulate the stiffness of the sensing mode, *k_B_*(*t*) = *k_B_* + Δ*k*(*q_A_*). Because the mode shape is two-fold symmetric, the stiffness change is insensitive to the sign of the displacement and Δ*k* approximates a rectified sine wave. The 2*ω* component of this rectified sine wave has a 45 degree phase shift relative to *q_A_*, establishing the phase relationship between the 2*ω* pump and 1*ω* signal waveforms (*F_C_* and *F_Q_*).

The resulting behavior can be understood as a degenerate parametric amplifier described by the Mathieu equation:
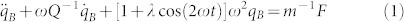
where *λ* = Δ*k*/*k_B_* is the fractional stiffness change. When *λ* = 0, the device is a linear resonator and the sensitivity to force at the resonance frequency *ω* is *Q*/*k*. When *λ* ≠ 0, the device is a parametric resonator and the excess parametric gain depends on the phase *ϕ* of the 1*ω* signal *F* relative to the 2*ω* pump^10^,
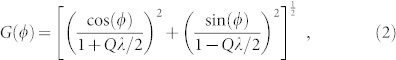
so that the total gain at resonance is given by *F*/*q* = *G*(*ϕ*)·*Q*/*k*. Maximum amplification occurs when the 1*ω* and 2*ω* signals are phase shifted by *ϕ* = 90°, and the system becomes self-oscillating when the stiffness change reaches a critical threshold, *λ_crit_* = 2*Q*^−1^, a condition known as autoparametric oscillation. Here, *Q* = 8 · 10^4^ and *λ_crit_* = 25·10^−6^, so even the very small nonlinear elastic behavior of the silicon disk results in significant parametric amplification, provided the two modes are degenerate. Experiments and finite element method (FEM) simulations (Supplementary Information) indicate that *λ_crit_* occurs at a vibration amplitude of 19 nm. Parametric amplification also affects the resonator's frequency response: the full-width at half-maximum (FWHM) of the resonance peak, Δ*ω*, which is proportional to *ωQ*^−1^ in a linear resonator, is reduced.

### Experimental Results

**Figure 4:**
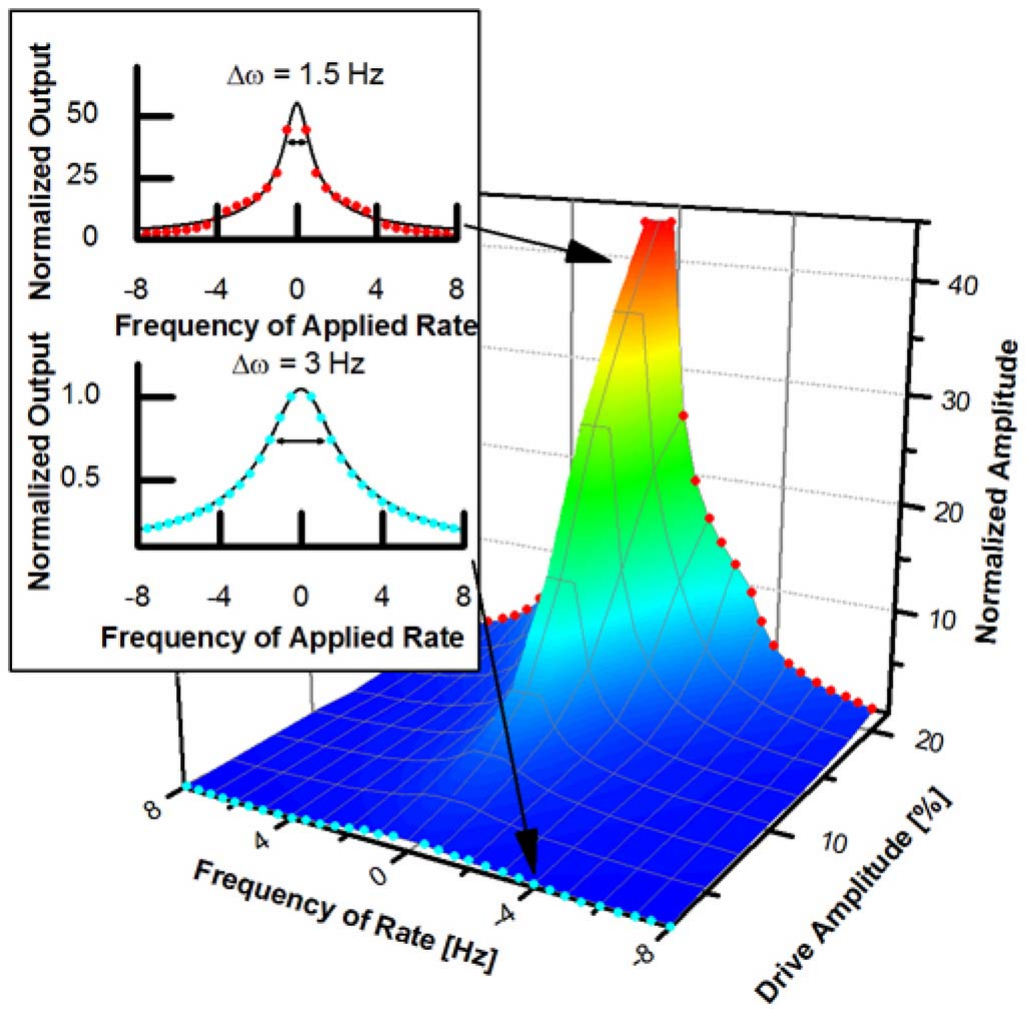
Observed response to rate. As the drive mode's amplitude is increased, the rate sensitivity increases nonlinearly, and quality factor *Q* is artificially increased, both resulting from self-induced parametric amplification of the Coriolis force. Inset shows the measured frequency response at small and large amplitudes, indicating the reduced bandwidth observed at large amplitude due to the artificial increase in *Q*.

Self-induced parametric amplification was first observed by measuring the gyroscope's sensitivity to rotation rate *S*_Ω_ as a function of the amplitude of the driven mode, 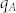. In what follows, we report 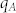 as a percentage of the capacitive electrode gap, *g* = 1.5 μm. Because electrostatic spring softening results in decreased resonant frequency of the drive axis at large 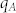 via the Duffing equation^34^, the electrostatic tuning voltage was adjusted at each 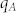 to maintain mode-match (*δω* ≈ 0) by finding the tuning voltage that maximized the sensitivity *S*_Ω_. Using a rate table, sinusoidal rotation rates with frequency varying from 0.2 Hz to 8 Hz were applied to the gyroscope. The resulting amplitude of the sensing mode, 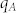, is plotted in Fig. 4. When the driven mode's amplitude is small 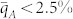, the frequency response exhibits the expected Lorentzian shape with Δ*ω*/2*π* = 3 Hz. As 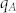 is increased, the scale-factor, *S*_Ω_, increases at a rate much greater than 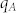; an 8-fold increase in 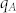 results in a 67-fold increase in *S*_Ω_ and a two-fold reduction in Δ*ω*.

**Figure 5:**
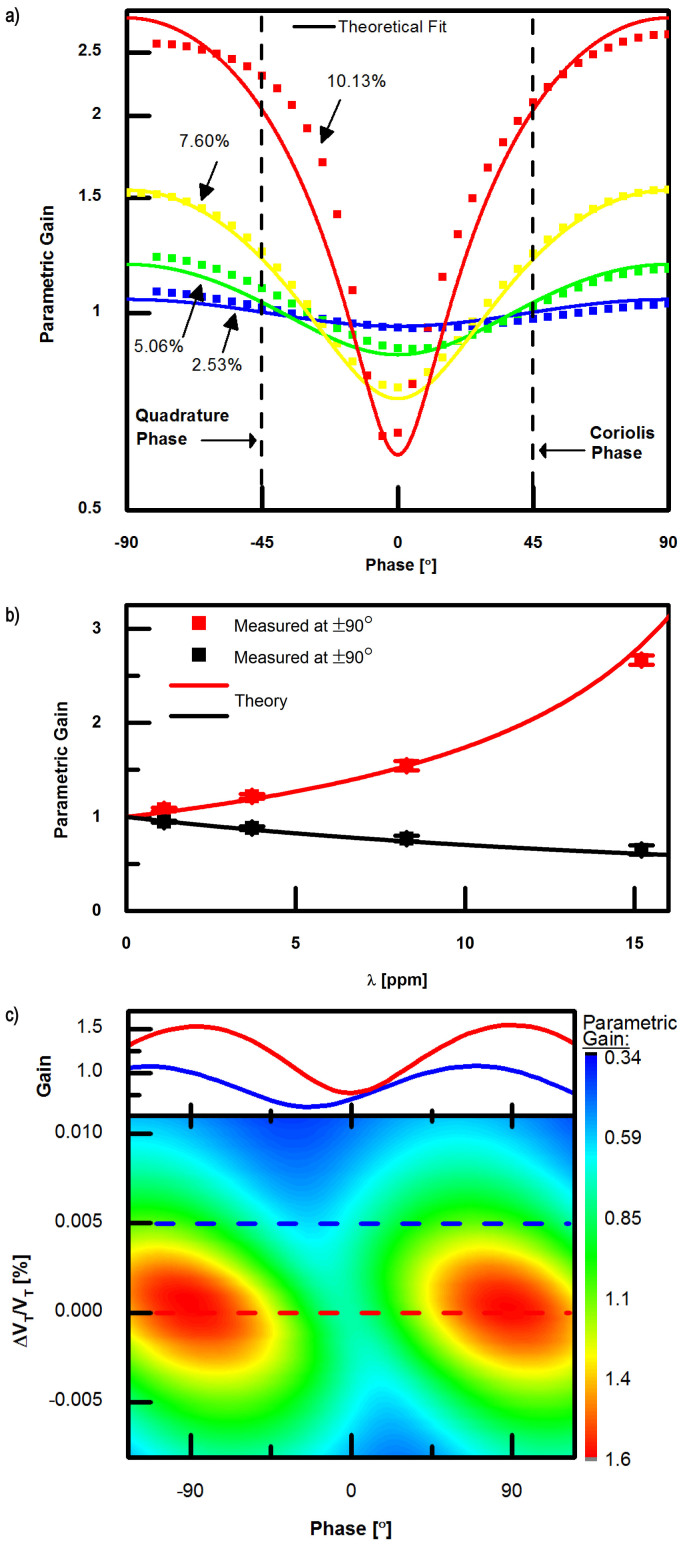
Electrostatically-probed parametric amplification. a) Parametric gain measured at various vibration amplitudes, 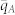, versus phase shift between the sense axis excitation force and the drive mode's vibration, with theoretical fit superposed. b) Measured and theoretical parametric gain at ±90° and 0° phase shifts versus change in stiffness extracted by fitting data in Fig. 5a) to Equation (2). c) Measured parametric gain at 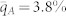 amplitude plotted as the tuning voltage is varied from V_T_, the voltage required for the degenerate (mode-matched) condition. At ΔV_T_ = 0 the modes are degenerate (red dashed line) and the parametric gain curve is symmetric about 0° phase shift, as shown in red in the top inset. Non-degenerate operation (blue dashed line), decreases the maximum gain and shifts the phase at which this occurs, shown in blue in the inset.

Due to the degeneracy of the two modes, which results in coupling between the two modes, the frequency shift of one mode induced by the motion of the other cannot be probed by standard techniques, such as those employed by Refs. 35, 36 and 37 which involve measuring the amplitude, phase, or frequency of the parametrically-amplified mode while the first mode is being excited either through a sweep or by locking to its resonance. In addition, much of the theory developed for predicting this behavior has been developed using Euler-Beam theory, and is not directly applicable to the complex geometry present in this device. Thus, in order to further characterize the observed amplification, the force sensitivity was probed by applying an additional electrostatic force directly to the sensing mode with a controlled phase relative to the excitation applied to the driven axis (Supplementary Information), and measuring the amplitude of the movement caused by this additional force. The baseline motion of the sensing mode due to modal coupling, electrode misalignment, and electrical feedthrough of the drive signal were subtracted, so that the resulting amplification of the force applied to the sense mode could be accurately measured. The system was allowed to stabilize for 10 seconds before measurements were taken to ensure a steady-state response, and the measurements themselves were averaged over a one-second interval. The resulting amplification of this additional force is shown in Fig. 5a, exhibiting phase dependence consistent with parametric amplification (Eq. 2), with maximum amplification occurring at *ϕ* = ±90° and minimum amplification at *ϕ* = 0°. The phase of the Coriolis and quadrature forces (*F_C_* and *F_Q_*) are indicated on the plot. We extract Δ*k* by fitting the experimental data with the theoretical model for *G*(*ϕ*), and the measured gain at 0° and 90° as a function of Δ*k* agrees well with the model, as shown in Fig. 5b. Finite element method (FEM) simulations that incorporate geometric stiffness nonlinearities yield values for Δ*k* that are similar in magnitude to the value extracted from experiments (Supplementary Information). Experiments conducted on resonators fabricated from silicon with two different dopants (n-type, Antimony, 2·10^18^ cm^−3^; p-type, Boron, 5·10^18^ cm^−3^) led to extracted Δ*k* values that were nearly identical, indicating that the observed nonlinearity is geometric in nature, rather than being due to intrinsic nonlinearity of the elastic coefficients of silicon, which are observed to depend strongly on dopant type and concentration^38^. The ac voltage used to actuate the drive mode of the DRG is a pure sinusoid at *ω*, but electrostatic nonlinearity of the transduction and tuning electrodes does contribute a small change in stiffness at 2*ω*^17^,^21^. A conservative estimate, however, shows this component to be no larger than 0.22 ppm even for very large amplitude vibration of the sensing mode (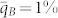) (Supplementary Information).

### Impact of Mode-Matched Operation

**Figure 6:**
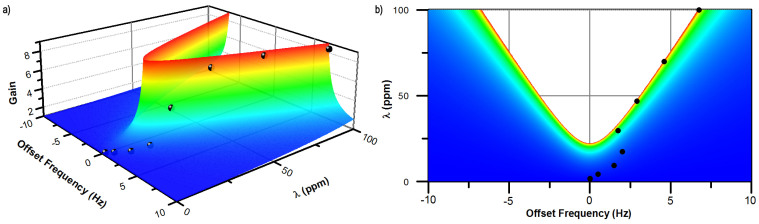
Theoretical parametric amplification of the Coriolis force versus frequency offset from 2*ω*. The measured amplification, black circles, is calculated by dividing the measured *S*_Ω_ by the linear prediction obtained from the first three data points. The measured points are plotted at the offset frequency required to obtain the observed amplification. a) Shows perspective view, and b) shows top-down view.

In this device, self-induced parametric amplification is a result of mode-matched operation. To demonstrate this, we investigated the effect of operating in a non-degenerate condition by adjusting the tuning voltage such that *δω* ≠ 0. Since the Δ*k* pump is at 2(*ω* + *δω*), mistuning results in diminished parametric gain, as demonstrated in Fig. 5c. Using *δ* = 4*k*/(*m*(2*ω* + *δω*)^2^) − 1 as a parameter to represent the normalized detuning, the parametric gain experienced by the Coriolis force is expected to be^39^
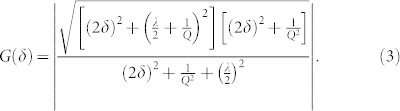
*G*(*δ*) is shown in Fig. 6 a and b, with the measured gain (calculated by dividing the measured *S*_Ω_ from Fig. 4 by the linear prediction) superposed. The stability boundary takes the shape of an Arnold's tongue^40^,^41^,^42^ as previously observed in nanomechanical systems^43^,^44^,^45^. The offset frequency for this data is selected so that the measured gain falls on the theoretical surface. Experimentally, this frequency offset is the result of tuning to maximize rate sensitivity: when *λ* exceeds *λ_crit_* for a given drive amplitude, the maximum sensitivity is obtained by introducing a frequency offset of several Hz, so that parametric amplification can be stably attained. Here, the maximum gain is limited to approximately 8. Larger parametric gains are difficult to achieve stably since they are quite sensitive to small variations in stiffness and quality factor, both of which depend on temperature.

## Discussion

The observed parametric gain, combined with operation at large drive displacements (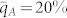), results in a 67-fold increase in sensitivity, as extracted by fitting to the measured data. This increased sensitivity can greatly reduce the impact of electronic noise from the readout electronics on the gyroscope's output, offering the potential to significantly reduce gyroscope power consumption. More fundamentally, it has been observed that mode-matched operation often results in greater instability of the gyroscope's zero-rate output, known as bias instability. Here, we observe that mode-matched operation introduces self-induced parametric amplification which, if un-regulated, results in dramatic sensitivity fluctuations which can lead to increased bias instability. Whether parametric amplification is intentionally employed to increase scale factor, or suppressed through electrostatic cancellation or design modifications to provide greater stability, knowledge of the presence of self-induced parametric amplification is critical to enable high-Q gyroscope operation at large drive amplitudes.

We present the first observation of self-induced parametric amplification due to nonlinear stiffness coupling between degenerate orthogonal vibration modes in a high quality-factor micromechanical resonator. This amplification has an important application to increase the rate sensitivity of vibratory gyroscopes and may find other applications in mechanical pre-amplification of other high quality factor micro- and nano-mechanical resonators.

## Methods

The DRG is fabricated in <100> silicon, and vacuum sealed via epitaxial encapsulation. The structure is 0.6 mm in diameter, and 40 μm thick. It consists of 36 concentric 3-μm thick rings with spokes spaced by 45° increments, and at alternating angles (offset by 22.5°) so that the structure is suspended from a single central anchor. The gaps between each ring and the transduction gap surrounding the structure are 1.5 μm. A bias voltage (15 V) is applied to the central anchor, so that movement of the structure induces a current on capacitive electrodes surrounding the structure. Additional electrodes surrounding the device are biased with tuning voltages (> −20 V) to achieve degenerate modes, *δω* ≈ 0.

The device is wirebonded out to a ceramic package and mounted on a printed circuit board (PCB), where the signals are amplified. A Zurich Instruments HF2LI lock-in amplifier is used to provide ac drive signals, bias, and tuning voltages, as well as to perform all the measurements described above.

The PCB with the gyro is mounted on a rate table for inertial testing. Although the rate table itself is not temperature-controlled, the device temperature is stabilized at approximately 28°C using a thermoelectric cooler (TEC) mounted below the PCB.

## Author Contributions

S.N. and M.L. conceived the experiments. S.N., M.L. and V.Z. developed supporting theory and performed experiments. V.Z. and S.N. performed finite element modeling. C.A. performed independent experiments, and fabricated the device. T.K., A.C. and D.H. oversaw the research, provided guidance, and discussed the results and implications at all stages. D.H. and S.N. wrote the manuscript, and all authors edited the manuscript.

## Supplementary Material

Supplementary Information Supplementary Information
