# Supplementary material for: Self-induced parametric amplification arising from nonlinear elastic coupling in a micromechanical resonating disk gyroscope
Source: Sci Rep. 2015 Mar 12;5:9036. doi: 10.1038/srep09036 (PMC4356982; doi:10.1038/srep09036)
Supplement: Supplementary Information [file srep09036-s1.pdf]

Self-induced parametric amplification arising from nonlinear elastic coupling in a micromechanical resonating disk gyroscope

Sarah H. Nitzan[1], Valentina Zega[2], Mo Li[1], Chae H. Ahn[3], Alberto Corigliano[2], Thomas W. Kenny[3], and David A. Horsley[1]

[1] University of California, Davis, CA, USA

[2] Politecnico di Milano, Milan, Italy

[3] Stanford University, Stanford, CA, USA

Supplementary Information

1. Epitaxial Encapsulation Process

Disk resonators are fabricated using an epitaxial silicon sealing process[1], co-developed between researchers at the Robert Bosch Research and Technology Center and Stanford University. The process results in a clean, low-pressure, oxide-free environment, without the use of getters that are commonly used in high vacuum packaging of MEMS resonators. Disk resonators are fabricated via deep reactive ion etching (DRIE) of the 40 µm-thick (100) single-crystal silicon device layer of a silicon-on-insulator (SOI) wafer. The etched trenches are filled with a 2 µm-thick sacrificial oxide layer, and electrical contact holes are etched into this oxide. The device is then sealed with an initial epitaxial silicon layer. Vent holes are etched into this layer to allow a vapor-phase hydrofluoric acid (HF) etch to remove the sacrificial oxide layer, releasing the resonator structure, after which a second epitaxial encapsulation layer is deposited to seal the vent holes and create the hermetic cavity. Low pressure annealing in a nitrogen environment is then performed to diffuse the residual hydrogen gas out of the cavity. A top aluminum metal layer provides electrical contact, after which the wafer is diced and individual resonator dice are wire-bonded into ceramic packages for testing.

2. Electrostatic Mode-Matching

The Coriolis coupling between the disk resonator's elliptical vibration modes is maximized when the two modes are degenerate. Imperfections and crystalline anisotropy[2] break the resonator's symmetry, splitting the frequency of the two modes so that they are not perfectly degenerate. The largest component of the frequency split ($\Delta f = \delta\omega/2\pi$ on the order of 1 kHz) is due to the anisotropic Young's modulus of <100> silicon and was compensated by making small

adjustments to the (nominally 45°) angle between the spokes that connect the resonator's concentric rings, as shown in Figure S1[3].

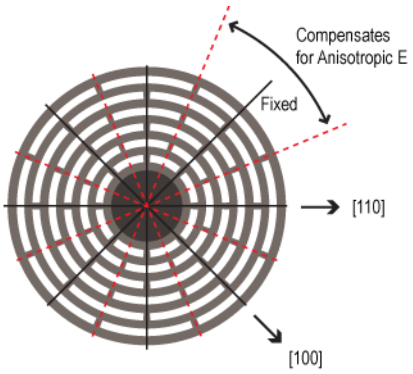

Figure S1: A cartoon showing the layout of the spokes and concentric rings that compose the disk resonator. Small adjustments to the angle between alternating spokes (shown in red) compensate for the anisotropy of Young's Modulus in <100> silicon.

The remaining Δf, on the order of 100 Hz – 300 Hz, originates from fabrication defects and must be nulled using electrostatic frequency tuning via the electrodes surrounding the device, which approximate parallel plate capacitors. The energy stored in a parallel plate capacitor operating in a vacuum is given by $W = V^2\varepsilon_0 A/2g$, where V is the applied voltage, ε_0 is the permittivity of free space, A is the electrode area, and $g = g_0 - q(t)$ is the gap between the electrode plates. The attractive force exerted by such an electrode is given by

$$F = \frac{\partial W}{\partial g} \approx \frac{\varepsilon_0 A}{2g_0^2}V^2 + \frac{\varepsilon_0 A}{g_0^3}V^2 q(t) \qquad \text{(S.1)}$$

for small $q(t)$. Thus, the capacitor acts as a spring with negative stiffness, $k = -V^2\varepsilon_0 A/g_0^3$. By adjusting the applied voltage, V, the stiffness, and therefore the frequency of a given vibration mode, can be tuned. In addition to matching the frequency of two resonant modes, this method can be used to minimize cross-coupling between the two modes that arises from off-diagonal elements of the stiffness matrix. This cross-coupling is the root cause of the quadrature force and occurs because the resonator's primary axis of stiffness is rotated with respect to the drive and

sense electrodes. A detailed discussion of electrode configurations for mode-matching of DRGs can be found in[4] and[5].

3. Modal Mass and Angular Gain

The modal mass of a given mode shape is calculated by equating the integrated strain energy, $W_k = \rho\int \dot{x}^2 dV = \rho\omega^2 \int x^2 dV,$ occurring at a given vibration amplitude, x, to the maximum kinetic energy, $m\dot{q}_A^2/2$, and solving for m. The resulting expression is

$$m = \frac{2W_k}{\omega^2 q^2} \tag{S.2}$$

where ω is the resonant frequency of the mode, and q is a reference displacement of the mode, chosen here to be the maximum radial displacement. The modal mass for this structure is 3.8 μg. The angular gain, determined by the mode shape, dictates what percentage of the drive mode's momentum is coupled to the sense mode by an applied Coriolis force. The angular gain is given by[6]

$$c = \iiint_V (\hat{r}_A \times \hat{r}_B) dV / \iiint_V (|\hat{r}_A|^2) dV \tag{S.3}$$

where A and B are the two modes under consideration, and $\hat{r}_A$ and $\hat{r}_B$ are the normalized displacement fields for each mode. The integrals are calculated over the volume V of the structure. For a tuning fork gyroscope, which translates linearly, $c = 1$. For a ring gyroscope or DRG operating in the 2θ mode, $c \approx 0.8$.

4. Linear Model for the Force Sensitivity of the Sense Axis

Using a linear model for the sense mode, the equation of motion is given by

$$m_B \ddot{q}_B + b_B \dot{q}_B + k_B q_B = F \tag{S.4}$$

where m_B, b_B and k_B are the modal mass, damping coefficient, and spring constant of the sense mode respectively. For this second-order harmonic oscillator, the quality factor is defined as $Q \equiv \sqrt{m_B k_B}/b_B$ and, for a harmonic force input at the resonant frequency ω, the force-to-displacement sensitivity is given by Qk_B^{-1}.

5. Device Operation and Electrostatic Testing

The block diagram of the disk resonator and associated control electronics is shown in Figure S2. Following electrostatic mode-matching, the driven mode is excited using a digital phase-locked loop (PLL) that is locked to the driven mode's natural frequency. The PLL amplitude is set to a constant value that is varied to achieve a desired vibration amplitude of the drive mode. Vibration of the sense mode is detected and subsequently quadrature demodulated using the PLL output as a reference. The in-phase component of the sense mode vibration is used as a measure of rotation rate, and the quadrature component is used to adjust the voltage on a dedicated set of electrodes used to null the quadrature signal.

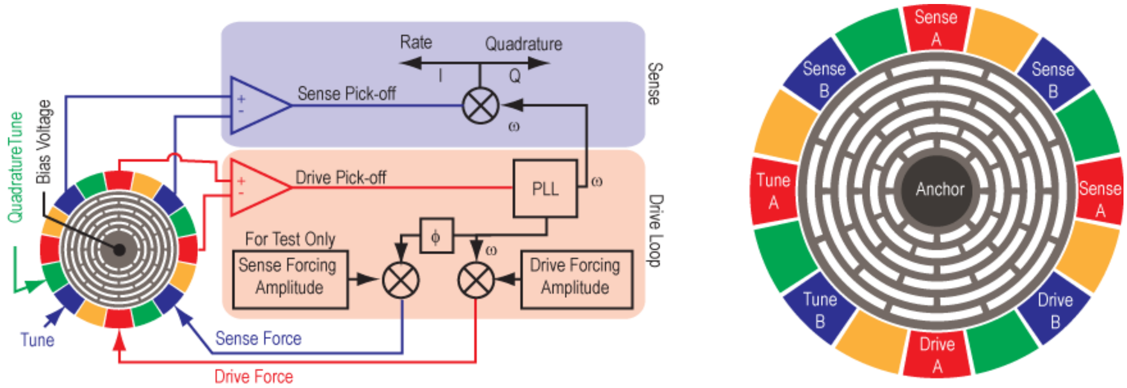

Figure S2: A block diagram of the disk resonator and associated control electronics is shown on the left, while the electrode layout and device design are shown on the right. Following electrostatic mode-matching, the drive mode is excited in a closed loop using a digital PLL. Vibration of the sense mode is demodulated into In-Phase (I) and Quadrature (Q) components, which contain the Coriolis force and quadrature force signals, respectively. The Q channel is used to adjust the voltage on a dedicated set of capacitive electrodes used for quadrature-nulling (shown in green and yellow).

To characterize spontaneous parametric amplification of the sense mode's response to a force with arbitrary phase ϕ relative to the drive mode's vibration, tests were conducted wherein a secondary electrostatic force was input to the sense mode, as shown in Figure S2. This testing configuration was used to generate the data shown in Figure 4.

6. Modeled and Experimental Change in Stiffness

Estimates for the fractional change in stiffness, $\lambda = \Delta k/k$, were extracted from experiments using two different techniques and compared to values estimated from a finite element method (FEM) model of the resonator. The first experimental technique was to extract λ from measurements of parametric amplification occurring in two different resonators having different doping (data presented in Fig. S3, below). The second experimental technique made use of the electrostatic stiffness modulation conventionally used to achieve parametric amplification in MEMS devices. Here, the *electrostatic* stiffness modulation was used to cancel the spontaneously-occurring *mechanical* stiffness modulation. An electrostatic pump signal at 2ω was applied to the capacitive electrodes of the sense mode and adjusted in amplitude until the observed spontaneous parametric amplification was cancelled. The relationship between the pump amplitude and the electrostatic stiffness (described in Section 2 above) was then used to provide a second estimate for λ.

FEM simulations were conducted using commercial software (COMSOL Multiphysics) to model the fractional stiffness change of the sense mode occurring at a given vibration amplitude of the drive mode. Because time-domain FEM simulations are computationally infeasible for this structure, an alternative method was developed. First a boundary load is applied which mimics the deformed mode shape, and the load amplitude required to produce a given deformation

amplitude was determined. With the resonator deformed using the determined boundary load, a prestressed nonlinear eigenfrequency analysis is then conducted to determine the eigenfrequency of the sense mode. The change in the eigenfrequency relative to the value from an unstressed eigenfrequency analysis provides an estimate of the sense mode's change in stiffness as a function of the drive mode's deformation amplitude. Fourier analysis is performed on the resulting function and the component at 2ω is λ.

The experimentally-extracted values for λ as a function of drive mode amplitude are presented along with the FEM predictions in Figure S3. Values extracted using both experimental techniques are in close agreement, whereas FEM slightly under-predicts the experimentally-observed values but shows the correct trend.

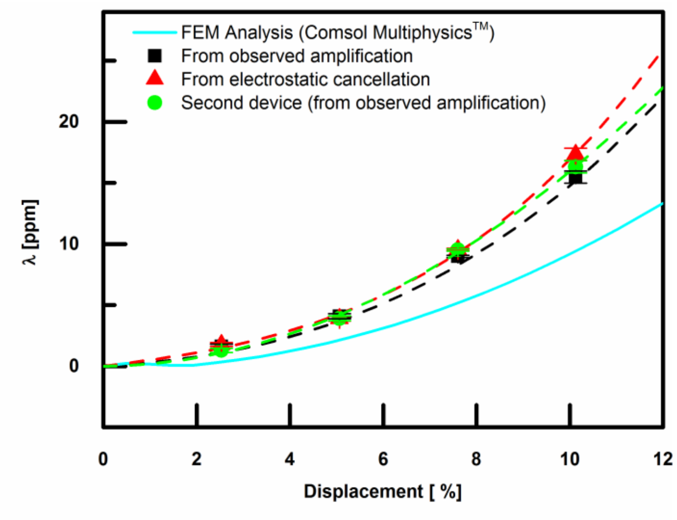

Figure S3: Modeled and experimentally-observed stiffness change at 2ω, $\lambda = \Delta k/k$, versus drive displacement. The change in stiffness was extracted from the observed parametric gain shown in Figure 3b (black squares) and by electrostatically cancelling the mechanically-induced stiffness change and extracting the corresponding λ (red triangles). The two approaches are in close agreement. A second device with different doping reveals the same λ versus displacement relationship, indicating that the source of the nonlinearity is geometric as opposed to intrinsic material nonlinearity. FEM estimates for λ under-predict the experimental values but show the correct trend.

7. Electrostatic Nonlinearity

The energy stored in an infinitesimal arc of a parallel plate electrode is given by

$$dW = \frac{\varepsilon_0 h}{2g(\theta)} V^2 r d\theta \tag{S.5}$$

where V is the applied voltage, ε_0 is the permittivity of free space, r is the radius, h is the height of the DRG, and $g(\theta) = g_0 - q(t,\theta)$ is the gap between the electrode plates. q is a function of time, t, and angular location, θ. The gap varies along the angular extent of the electrode in accordance with the vibration mode shape, $q(t,\theta) = \bar{q}\cos(\omega t)\cos(2\theta) = \bar{q}_t \cos(2\theta)$. The total energy of an electrode can therefore be expanded as

$$W = \frac{\varepsilon_0 r h}{2g_0} V^2 \int_{\psi-\alpha}^{\psi+\alpha} \left[1 + \frac{q}{g_o} + \left(\frac{q}{g_0}\right)^2 + \left(\frac{q}{g_0}\right)^3 + \left(\frac{q}{g_0}\right)^4 + \cdots\right] d\theta \tag{S.6}$$

where ψ is the angular location of the electrode center and 2α is the angular extent of the electrode[5]. The force exerted on the structure is then given by

$$F = \frac{\partial W}{\partial \bar{q}} = \frac{\varepsilon_0 r h}{2g_0} V^2 \int_{\psi-\alpha}^{\psi+\alpha} \left[\frac{\cos(2\theta)}{g_o} + 2\frac{\bar{q}_t \cos^2(2\theta)}{g_0^2} + 3\frac{\bar{q}_t^2 \cos^3(2\theta)}{g_0^3} + 4\frac{\bar{q}_t^3 \cos^4(2\theta)}{g_0^4} + \ldots\right] d\theta$$

(S.7)

which can be written as

$$F = F_0 - \bar{q}_t[k_1 + k_2(\bar{q}_t) + k_3(\bar{q}_t)]. \tag{S.8}$$

It can be seen that

$$k_3(\bar{q}_t) = -\bar{q}_t^2 \frac{\varepsilon_0 r h}{2g_0} V^2 \int_{\psi-\alpha}^{\psi+\alpha} \frac{4\cos^4(2\theta)}{g_0^4} d\theta$$

$$= -\bar{q}_t^2 \frac{\varepsilon_0 r h}{2g_0^5} V^2 [\cos(4\psi)\sin(4\alpha) + \frac{1}{8}\cos(8\psi)\sin(8\alpha) + 3\alpha] \tag{S.9}$$

Since $\bar{q}_t = \bar{q}\cos(\omega t)$, $\bar{q}_t^2$ has a 2ω component with amplitude $\bar{q}^2/2$, the change in stiffness at 2ω due to the nonlinearity of the transduction electrodes is given by

$$\Delta k_{2\omega} = \bar{q}^2 \frac{\varepsilon_0 r h}{4g_0^5} V^2 [\cos(4\psi)\sin(4\alpha) + \frac{1}{8}\cos(8\psi)\sin(8\alpha) + 3\alpha]. \tag{S.10}$$

Summing over all electrode locations and voltages, and assuming a very large quadrature displacement of 1% of the gap, yields $\Delta k_{2\omega}/k = \lambda = 0.22$ ppm. This value is an order of

magnitude smaller than the minimum value for λ required to explain the observed parametric amplification. Thus, we can safely conclude that the parametric amplification we observe is not due to the electrostatic nonlinearity of the sense electrodes.

1 Candler, R. N. *et al.* Long-Term and Accelerated Life Testing of a Novel Single-Wafer Vacuum Encapsulation for MEMS Resonators. *J. Micromech. Syst.* **15**, 1446-1456, doi:10.1109/JMEMS.2006.883586 (2006).

2 Hopcroft, M. A., Nix, W. D. & Kenny, T. W. What is the Young's Modulus of Silicon? *J. Micromech. Syst.* **19**, 229-238, doi:10.1109/JMEMS.2009.2039697 (2010).

3 Ahn, C. H. *et al.* Mode-Matching of Wineglass Mode Disk Resonator Gyroscope in (100) Single Crystal Silicon. *J. Micromech. Syst.*, in press, doi:10.1109/JMEMS.2014.2330590 (2014).

4 Su, T.-H. *et al.* Silicon MEMS Disk Resonator Gyroscope with an Integrated CMOS Analog Front-End. *Sensors Journal, IEEE*, vol.14, no.10, pp.3426,3432, Oct. 2014 doi: 10.1109/JSEN.2014.2335735

5 Gallacher, B. J. *et al.* Electrostatic correction of structural imperfections present in a microring gyroscope. *J. Micromech. Syst.* **14**, 221-234, doi:10.1109/JMEMS.2004.839325 (2005).

6 Cho, J. Y. *High-Performance Micromachined Vibratory Rate- and Rate-Integrating Gyroscopes* Ph. D. thesis, University of Michigan, (2012).
